# The Rice Rolled Fine Striped (RFS) CHD3/Mi-2 Chromatin Remodeling Factor Epigenetically Regulates Genes Involved in Oxidative Stress Responses During Leaf Development

**DOI:** 10.3389/fpls.2018.00364

**Published:** 2018-03-20

**Authors:** Sung-Hwan Cho, Chung-Hee Lee, Eunji Gi, Yehyun Yim, Hee-Jong Koh, Kiyoon Kang, Nam-Chon Paek

**Affiliations:** ^1^Department of Plant Science, Plant Genomics and Breeding Institute, Research Institute of Agriculture and Life Sciences, Seoul National University, Seoul, South Korea; ^2^Crop Biotechnology Institute, Institutes of Green Bio Science & Technology, Seoul National University, Seoul, South Korea

**Keywords:** chloroplast biogenesis, chromatin remodeling factor, leaf variegation, narrow leaf, reactive oxygen species, rice (*Oryza sativa*), Rolled Fine Striped

## Abstract

In rice (*Oryza sativa*), moderate leaf rolling increases photosynthetic competence and raises grain yield; therefore, this important agronomic trait has attracted much attention from plant biologists and breeders. However, the relevant molecular mechanism remains unclear. Here, we isolated and characterized *Rolled Fine Striped* (*RFS*), a key gene affecting rice leaf rolling, chloroplast development, and reactive oxygen species (ROS) scavenging. The *rfs-1* gamma-ray allele and the *rfs-2* T-DNA insertion allele of *RFS* failed to complement each other and their mutants had similar phenotypes, producing extremely incurved leaves due to defective development of vascular cells on the adaxial side. Map-based cloning showed that the *rfs-1* mutant harbors a 9-bp deletion in a gene encoding a predicted CHD3/Mi-2 chromatin remodeling factor belonging to the SNF2-ATP-dependent chromatin remodeling family. *RFS* was expressed in various tissues and accumulated mainly in the vascular cells throughout leaf development. Furthermore, *RFS* deficiency resulted in a cell death phenotype that was caused by ROS accumulation in developing leaves. We found that expression of five ROS-scavenging genes [encoding catalase C, ascorbate peroxidase 8, a putative copper/zinc superoxide dismutase (SOD), a putative SOD, and peroxiredoxin IIE2] decreased in *rfs-2* mutants. Western-blot and chromatin immunoprecipitation (ChIP) assays demonstrated that *rfs-2* mutants have reduced H3K4me3 levels in ROS-related genes. Loss-of-function in *RFS* also led to multiple developmental defects, affecting pollen development, grain filling, and root development. Our results suggest that RFS is required for many aspects of plant development and its function is closely associated with epigenetic regulation of genes that modulate ROS homeostasis.

## Introduction

In eukaryotes, the organization of chromatin compacts DNA within the nucleus and plays a central role in regulating transcription by controlling the access of DNA to the transcriptional machinery (Kwon and Wagner, 2007; Li et al., 2007). Chromatin structure can be altered through DNA methylation, histone modification, and ATP-dependent chromatin remodeling (Kwon and Wagner, 2007; Li et al., 2007; Shen and Xu, 2009; Ho and Crabtree, 2010). Chromatin remodeling disrupts DNA–protein interactions, altering the accessibility of specific DNA regions to regulatory proteins in the transcriptional machinery (Zhang K. et al., 2007; Clapier and Cairns, 2009).

Among chromatin-remodeling proteins, the Chromodomain Helicase DNA-binding (CHD) proteins have several conserved domains: one or two plant homeodomain (PHD) fingers, a chromodomain, a SNF2 (Sucrose Non-Fermenting)-related helicase/ATPase domain, and a DNA-binding domain (Aasland and Stewart, 1995). CHD proteins are important regulators of gene expression and participate in many development processes. Several *Arabidopsis thaliana* CHD members have been characterized. Null mutation of the SNF2-class ATPase SPLAYED (SYD) resulted in reduction of *WUSCHEL* (*WUS*) expression and decreased shoot apical meristem size (Kwon et al., 2005). SYD protein directly binds to the *WUS* promoter, supporting the notion that ATP-dependent chromatin remodeling directly regulates *WUS* activation (Kwon et al., 2005).

Another chromatin-remodeling protein, PICKLE (PKL), a putative SNF2-like ATPase subunit, activates embryo development and regulates transcript levels of genes specifically expressed in the embryo, such as *LEAFY COTYLEDON 1* (*LEC1*), *LEC2*, *FUSCA3* (*FUS3*), and *PHERES1* (Ogas et al., 1997, 1999; Dean Rider et al., 2003; Li et al., 2005). Moreover, PKL affects the expression of genes that respond to hormones. For example, during germination, PKL regulates gibberellin-modulated developmental programs to prevent re-expression of the embryonic developmental state (Ogas et al., 1999; Hay et al., 2002). PKL is required for repression of *AUXIN RESPONSE FACTOR 7* (*ARF7*) and *ARF9*, which encode auxin-responsive transcription activators; this repression is mediated by SOLITARY-ROOT (SLR)/IAA14 (Fukaki et al., 2006).

In rice, previous studies have reported that *OsCHR4/CHR729*, which encodes a CHD3/Mi-2 chromatin-remodeling factor, affects many aspects of plant development. Null mutation of *OsCHR4* caused defects in chloroplast development in adaxial mesophyll cells (Zhao et al., 2012). Moreover, *chr729* mutants exhibited rolled leaves with white stripes on the adaxial side, reduced stem elongation, and decreased chlorophyll contents (Hu et al., 2012). CHR729 plays important roles in seedling and root development via the gibberellin and auxin-related signaling pathways, respectively (Ma et al., 2015; Wang et al., 2016). In addition, CHR729 recognizes the histone modifications H3K4me3 and H3K27me3 and modulates their levels to control target genes involved in plant development (Hu et al., 2012). These observations suggest that chromatin-remodeling factors such as CHD proteins, PKL, and CHR729 may be involved in several aspects of plant development.

Reactive oxygen species (ROS) play key roles as signaling molecules in plant development and defense, but excess ROS damages cells. Low levels of ROS are continuously produced as byproducts of various metabolic pathways, such as the electron transport chain in chloroplasts and mitochondria, and photorespiration in peroxisomes (Dat et al., 2000). However, various abiotic stresses can lead to overproduction of ROS, which damage proteins, lipids, carbohydrates, and DNA, resulting in cell death due to oxidative stress (Polle, 2001; Mittler et al., 2004). Therefore, cells must maintain ROS homeostasis by removing excess ROS and regulating the anti-oxidative defense machinery, including ROS-scavenging enzymes (Noctor and Foyer, 1998; Apel and Hirt, 2004; Mittler et al., 2004). Soils containing alkaline salts (NaHCO_3_ and Na_2_CO_3_), an abiotic stress that can trigger an ROS burst, adversely affect plant growth and development and threaten crop productivity (Shi and Yin, 1993; FAO/AGL, 2000). In rice, functional deficiency of ALT1 (ALkaline Tolerance 1), a Snf2 family chromatin-remodeling ATPase, reduced the ROS levels and thus alleviated oxidative damage, resulting in improved tolerance to alkaline stress (Guo et al., 2014).

Here, we report the functional characterization of *RFS*, which encodes a predicted CHR4/Mi-2-like chromatin remodeling factor that regulates rice leaf rolling, leaf width, and chloroplast development. Functional deficiency of RFS causes accumulation of ROS due to reduced expression of ROS-scavenging genes, resulting in chlorotic phenotypes in *rfs* mutants. Mutation of *RFS* leads to the loss of H3K4me3 at the genomic loci of ROS-related genes, including *CATC, APX8*, and *Prx IIE2*, and loci encoding a putative copper/zinc SOD and a putative SOD. Our data provide strong, novel evidence that chromatin remodeling by RFS plays an important role in epigenetic regulation of ROS-related genes and plant morphogenesis.

## Materials and Methods

### Plant Materials and Growth Conditions

The *rfs-1* mutant, FL233 (Iwata et al., 1984), was induced in the rice *japonica* cultivar Norin-8 by gamma-ray mutagenesis. The *rfs-2* mutant line, T-DNA-insertion mutant PFG 3D-02766, was obtained from the Crop Biotech Institute at Kyung Hee University, South Korea (Jeon et al., 2000; Jeong et al., 2006). The *rfs-1* mutant was crossed with the Korean *japonica* rice cultivar ‘Seolakbyeo’ and progressed to the F_6_ generation. ‘Seolakbyeo’ and “Dongjinbyeo” were used as the parental WT plants for *rfs-1* and *rfs-2* mutants in this study. The *rfs-1* and *rfs-2* mutants were screened under natural long-day (LD) conditions in a paddy field (Suwon, South Korea, 37° N latitude). The plants in the growth chambers were grown under LD conditions: 14-h light (300 μmol m^-2^ s^-1^), 30°C/10-h dark, 28°C.

### Identification of Homozygous *rfs-2* Mutants From the T-DNA Tagged Line

Homozygous progeny of *rfs-2* T-DNA-tagged mutants were screened from T_1_ plants of the mutants by PCR. Genotyping was performed with 33 cycles of 95°C for 15 s, 58°C for 30 s, and 72°C for 45 s, using a combination of specific and T-DNA border primers as follows. The primer sets for *rfs-2* included left primer (LP), right primer (RP), and border primer (BP), as shown in Supplementary Table S1. BP and RP were used to obtain the T-DNA flanking sequences by PCR.

### Measurement of Chlorophyll Contents

Leaf blades of 2-month-old plants were frozen with liquid nitrogen and then ground into powder with a mortar and pestle. Then the leaf tissues were homogenized and resuspended in pre-chilled 80% (v/v) acetone. Residual plant debris was removed by centrifugation. The supernatants of these samples were then analyzed with a spectrophotometer; the concentrations of chlorophylls and carotenoids were measured at 663.2, 646.8, and 470 nm, and pigment concentrations were determined using the method of Lichtenthaler (1987).

### ROS Analyses

Detection of H_2_O_2_, O_2_^-^, and ^1^O_2_ by staining of leaves with DAB, NBT, and SOSG, respectively, was conducted according to published protocols (Driever et al., 2009). For DAB and NBT staining, the leaf blades of 2-month-old plants were infiltrated with 10 mM MES (pH 6.5) buffer containing 0.1% (w/v) DAB or 50 mM sodium phosphate buffer containing 0.05% (w/v) NBT for 8 h in darkness, then leaves were placed in boiling water for 20 min. The samples were soaked in 96% ethanol at room temperature for 8 h. Afterward, the cleared leaves were preserved in 50% ethanol and photographed. For SOSG imaging, the leaves of 2-month-old WT and *rfs-1* plants were vacuum infiltrated with 100 μM SOSG in the dark. SOSG fluorescence images were taken with a confocal laser scanning microscope (Carl Zeiss LSM710).

### Histological GUS Analysis

For the GUS assay, the 1893-bp genomic promoter fragment with one end in the first exon of the *RFS* gene, including the translation start codon, was PCR amplified (primers shown in Supplementary Table S1). Using the Gateway system (Invitrogen), the promoter fragment was cloned into the pENTR/D-TOPO vector, then an LR recombination reaction was performed with the destination vector pMDC162 to obtain the GUS reporter construct (*Pro_RFS_:GUS*). The plasmid was transformed into the rice *japonica* cultivar “Dongjinbyeo,” and the resulting transgenic plants were analyzed by GUS staining as described (Cho et al., 2016). Histological analysis was conducted according to a previously published method (Cho et al., 2013). The leaf samples of 12-week-old transgenic plants grown in a paddy field were fixed in formaldehyde–acetic acid solution (50% ethanol, 5% acetic acid, and 3.7% formaldehyde) for 1 day at 4°C, and dehydrated in an ethanol series (50, 75, 85, 95, and 100%), cleared through a xylene series (25, 50, 75, 90, and 100%), then infiltrated through a series of Paraplast Plus (Sigma), and finally embedded in 100% Paraplast at 55 ∼ 60°C. Then, 10- to 12-μm-thick microtome sections were mounted on glass slides. The sections were deparaffinized in 100% xylene and dried before staining with Toluidine blue O (Sigma). The deparaffinized samples were mounted in Permount (Fisher Scientific) with a cover glass. The cross sections were observed with a light microscope (Olympus BX50) at 10–100× magnification.

### Transmission Electron Microscopy

Transmission electron microscopy was conducted according to a previously published method (Yoo et al., 2009). Leaf samples of 12-week-old plants grown in a paddy field were harvested and fixed with Modified Karnovsky’s fixative and washed three times in sodium cacodylate buffer. Then, the samples were post-fixed in osmium tetroxide in sodium cacodylate buffer and briefly washed two times in distilled water. They were en-bloc stained in uranyl acetate overnight, dehydrated in a graded ethanol series, washed two times in propylene oxide, and then embedded in Spurr’s resin. After polymerization, ultrathin sections were cut with a diamond knife on an ultramicrotome (MT-X; RMC, Tucson, AZ, United States) and mounted on Formvar-coated copper grids. The sections on the grids were stained with uranyl acetate and Reynolds’ lead citrate, and then examined with an electron microscope (JEM-1010 EX; JEOL, Japan).

### Scanning Electron Microscopy

Leaf segments of 12-week-old plants were fixed with Modified Karnovsky’s fixative (2% paraformaldehyde and 2% glutaraldehyde in 0.05 M sodium cacodylate buffer at pH 7.2) for 4 h and washed three times in 0.05 M sodium cacodylate buffer (pH 7.2) at 4°C for 10 min. Then, the samples were post-fixed in 1% osmium tetroxide in 0.05 M sodium cacodylate buffer (pH 7.2) at 4°C for 2 h and briefly washed two times in distilled water at room temperature (as described for transmission electron microscopy). Samples were dehydrated in a graded ethanol series (EtOH 30, 50, 70, 80, 90, 100, 100, and 100%). Then, specimens were dried two times in 100% hexamethyldisilazane for 15 min and mounted on metal stubs. After mounting, samples were coated with gold and then observed with a scanning electron microscope (JSM-5410LV; JEOL, Tokyo, Japan).

### Map-Based Cloning

To identify the *RFS* gene using a map-based cloning approach, *rfs-1* was crossed to Milyang23, a Tongil-type cultivar that has a genetic makeup similar to that of *indica*, to generate a large F_2_ mapping population. For the fine mapping of the *rfs-1* locus, 755 F_3_ progeny were further generated from F_2_ heterozygous lines. PCR-based markers on chromosome 7 were developed based on the sequence difference between *japonica* and *indica* varieties in GRAMENE^1^ and the Arizona Genomics Institute^2^. For the physical mapping of *rfs-1*, four sequence-tagged site (STS) markers and one simple sequence repeat (SSR) marker and were used in AP003956 and AP004259, respectively. The primer sequences for the molecular markers used are listed in Supplementary Table S1.

### Complementation Test

For the complementation test, *rfs-1* was crossed to *rfs-2*, a T-DNA insertion mutant line, and then the phenotype of F_1_ progenies was analyzed in a paddy field.

### Isolation of Total RNA and RT-qPCR Analysis

Total RNA was extracted from 2-month-old plants that were grown in the growth chamber using the Total RNA Extraction Kit (MGmed, South Korea) according to the manufacturer’s manual. First-strand cDNAs were obtained from 2 μg total RNA using M-MLV reverse transcriptase and oligo(dT)_15_ primers (Promega). RT-qPCR analysis was performed in a 50-μl mixture containing 1/100th volume of cDNA preparation and GoTaq qPCR Master Mix (Promega). The primer sets used in this study are listed in Supplementary Table S1. The qPCR amplification was performed on a LightCycler 480 (Roche) using the following conditions: 2 min at 95°C followed by 50 cycles of 95°C for 30 s, 55°C for 30 s, and 72°C for 30 s. Relative expression levels of each gene were normalized to the transcript levels of a housekeeping gene *OsUBQ5* using the 2^-ΔΔ^*^C^*_T_ method (Rao et al., 2013), where *C*_T_ is the threshold cycle for each gene in every sample. The primer sequences for RT-qPCR are listed in Supplementary Table S1.

### Histone Protein Extraction and Immunoblot Analysis

Histone proteins were extracted from the leaf tissues of 2-week-old plants as previously described (Tariq et al., 2003). After being washed in acetone and dried, the proteins were resuspended in Laemmli sample buffer (62.5 mM Tris-HCl, pH 6.8, 2% SDS, 25% glycerol, 0.01% bromophenol blue, and 10% β-mercaptoethanol), then separated on a 15% SDS-PAGE and transferred to an Immobilon-P PVDF transfer membrane (Millipore). The membrane was blocked with 2% bovine serum albumin in phosphate-buffered saline (pH 7.5), and incubated overnight with primary antibodies against dimethyl-histone H3K9, trimethyl-histone H3K4, trimethyl-histone H3K27, and histone H3 (Millipore; catalog nos. 07–441, 07–473, 07–449, and 06–775, respectively) at a 1:5,000 dilution at room temperature. After three washes (30 min each), the secondary antibody [goat anti-rabbit IgG (Southern Biotech)] at a 1:10,000 dilution was used. For immunoblotting detection, we used an enhanced chemiluminescence detection system (WESTSAVE-Up; AbFrontier). The intensities of each protein were calculated using Image J (version 1.51s) software according to the instructions^3^. The levels of each protein are shown relative to those of WT, which is set as 1, and are indicated on the bottom of each protein blot.

### Chromatin Immunoprecipitation (ChIP) Assay

Leaf blade tissues of 2-week-old plants were collected for ChIP experiments as previously described (Kim et al., 2015). Briefly, chromatin isolated from 1 g of rice leaves was crosslinked in 1% formaldehyde solution. Chromatin complexes isolated with lysis buffer [50 mM Tris-HCl (pH 8.0), 10 mM EDTA, 1% SDS, 1 mM PMSF, protease inhibitor cocktail] were incubated with antibody-coated beads (Millipore, Cat. No. 16–157) overnight. After washes and elution, the products were reverse crosslinked. Then the products were treated with proteinase K (Sigma, 03115887001), recovered, and used as a template for RT-qPCR with primers listed in Supplementary Table S1. The antibodies for histone modification anti-H3K4me3 were from Millipore, Cat. No. 07–473.

### Accession Numbers

Sequence data from this article can be found in GenBank/EMBL databases under the following accession numbers: *RFS* (Os07g31450), *CATA* (Os02g02400), *CATB* (Os06g51150), *CATC* (Os03g03910), *APX1* (Os03g17690), *APX2* (Os07g49400), *APX3* (Os04g14980), *APX4* (Os08g43560), *APX5* (Os12g07830), *APX6* (Og12g07820), *APX7* (Os04g35520), *APX8* (Os02g34810), putative Cu/Zn-SOD (Os03g11960), *SodCc1* (Os03g22810), putative Cu-SOD (Os04g48410), *SodA1* (Os05g25850), putative SOD (Os06g02500), Fe-SOD (Os06g05110), *SodCc2* (Os07g46990), plastidic Cu/Zn-SOD (Os08g44770), *RbohF* (Os08g35210), *Prx IIE2* (Os02g09940), *OsUBQ5* (Os01g22490), *OsActin7* (Os11g06390).

## Results

### The *rfs-1* Mutant Exhibits Rolled Leaf Blades With Fine Stripes and Reduced Leaf Width

**FIGURE 1:**
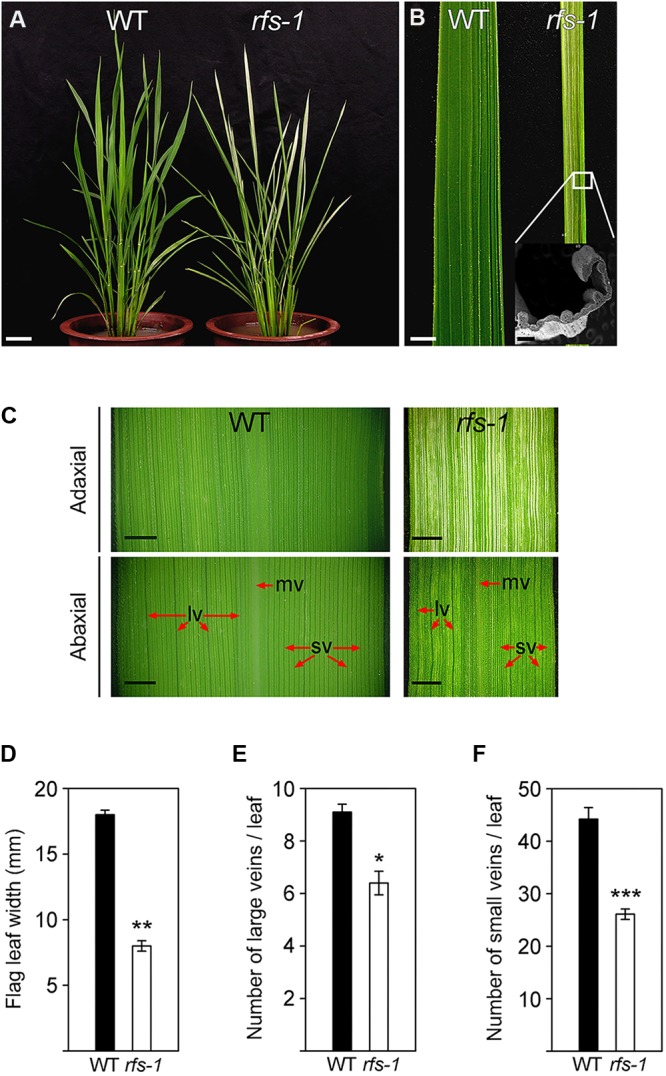
The *rfs-1* mutant has irregular white stripes on the leaf blades. **(A)** Phenotypes of 2-month-old WT and *rfs-1* plants. **(B)** Rolled-leaf phenotype in the *rfs-1* mutant. **(C)** Adaxial and abaxial sides of WT and *rfs-1* leaves. Midrib vein (mv); large vein (lv); small vein (sv). **(D)** Flag leaf width of WT and *rfs-1*. **(E)** Number of large veins in WT and *rfs-1* flag leaves. **(F)** Number of small veins in WT and *rfs-1* flag leaves. Scale bars: 5 cm **(A)**; 5 mm **(B)**; 2 mm **(C)**. Error bars indicate ± SD (*n* = 10). Asterisks indicate statistically significant differences compared to WT, as determined by Student’s *t*-test (^∗^*P* < 0.05, ^∗∗^*P* < 0.01, ^∗∗∗^*P* < 0.005).

The *rfs-1* mutant was named for its visible phenotype of rolled leave with fine stripes and was isolated in the M_6_ plants from a cross of the Korean *japonica* rice cultivar ‘Seolakbyeo’ with a gamma-ray-treated line of *Oryza sativa* ssp. *japonica* ‘Norin 8’. The *rfs-1* mutant had reduced leaf width, leaves that rolled to the adaxial side, and variegated leaves, which appeared in the 2-month-old plants (**Figures 1A–C**). To compare the leaf width of the *rfs-1* mutant with WT, we measured the maximum widths of flag leaves, approximately at the midpoint, which revealed that the *rfs-1* mutants have significantly narrower leaf blades with approximately 42% of the width of the WT (**Figures 1C,D**). In addition, the distance between longitudinal veins in *rfs-1* leaves was significantly smaller than in WT (**Figure 1C**). To ascertain whether the *rfs-1* mutants had fewer leaf veins, we counted the large and small veins per flag leaf at the midpoint of the leaf blade. The *rfs-1* mutants had fewer large and small veins, approximately 68 and 59%, respectively, of the number observed in WT (**Figures 1E,F**). Therefore, null mutation of *RFS* reduced the leaf width and the number of leaf veins.

### The *rfs-1* Mutants Have Defects in Vascular Bundle and Bulliform Cell Development

**FIGURE 2:**
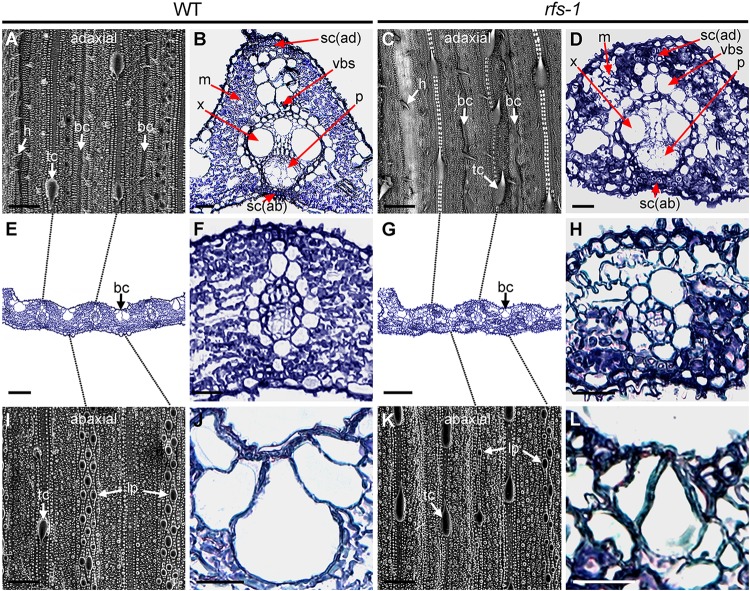
The *rfs-1* mutants show defective vascular development in leaves. **(A,C)** Scanning electron micrograph of the WT and *rfs-1* adaxial epidermis. bulliform cell (bc); hair (h); trichome (tc). **(B,D)** Transverse section of WT and *rfs-1* large vein. sc (ad), adaxial sclerenchyma; vbs, vascular bundle sheath; m, mesophyll cell; x, xylem; p, phloem; sc (ab), abaxial sclerenchyma. **(E,G)** Transverse section through WT and *rfs-1* small veins. Bulliform cell (bc). **(F,H)** Enlarged transverse section of WT and *rfs-1* small veins. **(I,K)** Scanning electron micrograph of the WT and *rfs-1* abaxial epidermis. large papilla (lp); trichome (tc). **(J,L)** Enlarged transverse section of WT and *rfs-1* bulliform cells. Scale bars: 100 μm **(A,C,E,G,I,K)**; 50 μm **(B,D,F,H,J,L)**.

The leaves of *rfs-1* mutants curved toward the adaxial side at the seedling stage and formed a cylinder-like shape at the mature stage. We cross-sectioned the leaves and performed scanning electron microscopy using the mature flag leaves of WT and *rfs-1* to investigate whether the *rfs-1* leaf rolling was due to changes in vascular bundle and bulliform cells. In WT, vascular bundle and bulliform cells typically developed large and small veins (**Figures 2B,E,F**), whereas the vascular bundle and bulliform cells of *rfs-1* mutants were not fully developed (**Figures 2D,G,H**). On the adaxial side, the average vascular bundle cells were small and poorly differentiated in the *rfs-1* mutants. Furthermore, the WT bulliform cells were well arranged in groups of five cells, with the middle cells being larger than the surrounding bulliform cells (**Figure 2J**). The *rfs-1* mutants had groups of 3 or 4 bulliform cells that were smaller than WT and irregular in shape and size (**Figure 2I**).

We also investigated the surface of mature WT and *rfs-1* leaves. In the adaxial epidermis of flag leaves, the WT leaves were relatively smooth and regular, with straight rows of bulliform cells, and had hairs on the sides of large and small veins (**Figure 2A**). By contrast, the *rfs-1* mutants had a distorted arrangement of bulliform cells and fewer hairs on the side of both veins (**Figure 2C**). In the abaxial epidermis of flag leaves, WT had round and regularly arranged large papillae (**Figure 2I**), whereas the large papillae of *rfs-1* mutants were significantly smaller and some were absent (**Figure 2K**). These results suggest that the adaxial side leaf rolling in the *rfs-1* mutants was affected by the irregular and distorted shapes of the vascular bundle and bulliform cells. Thus, because it is obvious that RFS is involved in development of the vasculature during leaf development, further epigenetic regulation of leaf development-related genes by RFS, will provide new insights into how RFS controls leaf development.

### The *rfs-1* Mutant Has Defects in Chloroplast Morphogenesis

The WT leaves were green on both adaxial and abaxial sides, whereas the *rfs-1* leaves showed a white-striped phenotype only on the adaxial side (**Figure 1C**). To investigate the chloroplast development of *rfs-1* mutants in more detail, we examined the chlorophyll contents, the density of mesophyll cells, and the ultrastructure of the chloroplasts. The amounts of chlorophylls and carotenoids were about 48% lower in the *rfs-1* mutants than in WT (Supplementary Figure S1C).

To determine whether the anatomical and morphological alterations detected in the *rfs-1* mutants were accompanied by ultrastructural changes, the leaf tissues of WT and *rfs-1* were examined by confocal microscopy and transmission electron microscopy. Confocal microscopic observation indicated that mesophyll cells on the adaxial side of *rfs-1* leaves had abnormal densities and distribution (Supplementary Figures S1A,B). The WT chloroplasts had well-developed starch granules and grana (Supplementary Figures S1D,E). The chloroplasts in the green sectors of *rfs-1* leaves had less well-ordered stacked grana and thylakoid structure and contained a few small plastoglobules (Supplementary Figures S1F,G).Moreover, the white sectors of *rfs-1* leaves contained undifferentiated and defective chloroplasts with much larger plastoglobules and small vacuole-like structures (Supplementary Figures S1G–I). The nucleus was the only normal organelle in the *rfs-1* leaf cells (Supplementary Figure S1H). These results demonstrate that defective chloroplasts in the *rfs-1* mutants were due to not only arrest of chloroplast biogenesis but also degeneration of chloroplasts during leaf development.

### Reactive Oxygen Species Accumulated in the *rfs* Leaves

**FIGURE 3:**
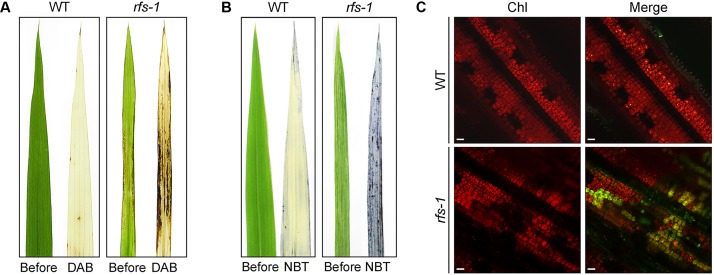
The *rfs-1* mutant accumulates more ROS in the leaves. **(A,B)** DAB staining for hydrogen peroxide (H_2_O_2_) (dark brown, **A**) and NBT staining for superoxide (O_2_^-^) (blue, **B**) in the leaf blades of 2-month-old WT and *rfs-1*. Before, leaf blade prior to staining; DAB, cleared leaf after DAB staining; NBT, cleared leaf after NBT staining. **(C)** Visualization of singlet oxygen (^1^O_2_) detected with the SOSG fluorescent probe on the leaves of 2-month-old WT and *rfs-1*. The fluorescence of SOSG is shown in green; the red color corresponds to chlorophyll (Chl) auto-fluorescence. Scale bars: 10 μm **(B)**. DAB, 3,3′-diaminobenzidine; NBT, nitroblue tetrazolium; SOSG, singlet oxygen sensor green.

The overproduction of ROS in plants can cause chlorosis and cell death (Polle, 2001; Mittler et al., 2004; Ye et al., 2016). If the *rfs-1* mutant is defective in ROS homeostasis, it may be incapable of efficiently inducing the ROS-scavenging system. To investigate whether the chlorotic and cell death phenotypes that were observed in the adaxial side of *rfs-1* leaves were caused by the accumulation of ROS, we stained for hydrogen peroxide (H_2_O_2_) and superoxide radical (O_2_^-^) using DAB and NBT, respectively. The leaf blades of 2-month-old *rfs-1* mutants exhibited a chlorotic phenotype and stained more strongly than those of WT (**Figures 3A,B**). Moreover, singlet oxygen (^1^O_2_) imaging was performed by staining the leaves with the ^1^O_2_-specific fluorescent dye SOSG. The *rfs-1* leaves showed much more intense staining with SOSG than the WT leaves (**Figure 3C**). These results suggested that the accumulation of ROS caused the chlorotic and cell death phenotypes in the *rfs-1* mutants, presumably due to the lack of proper ROS detoxification.

### RFS Encodes a CHR4/Mi-2-Like Chromatin Remodeling Factor

**FIGURE 4:**
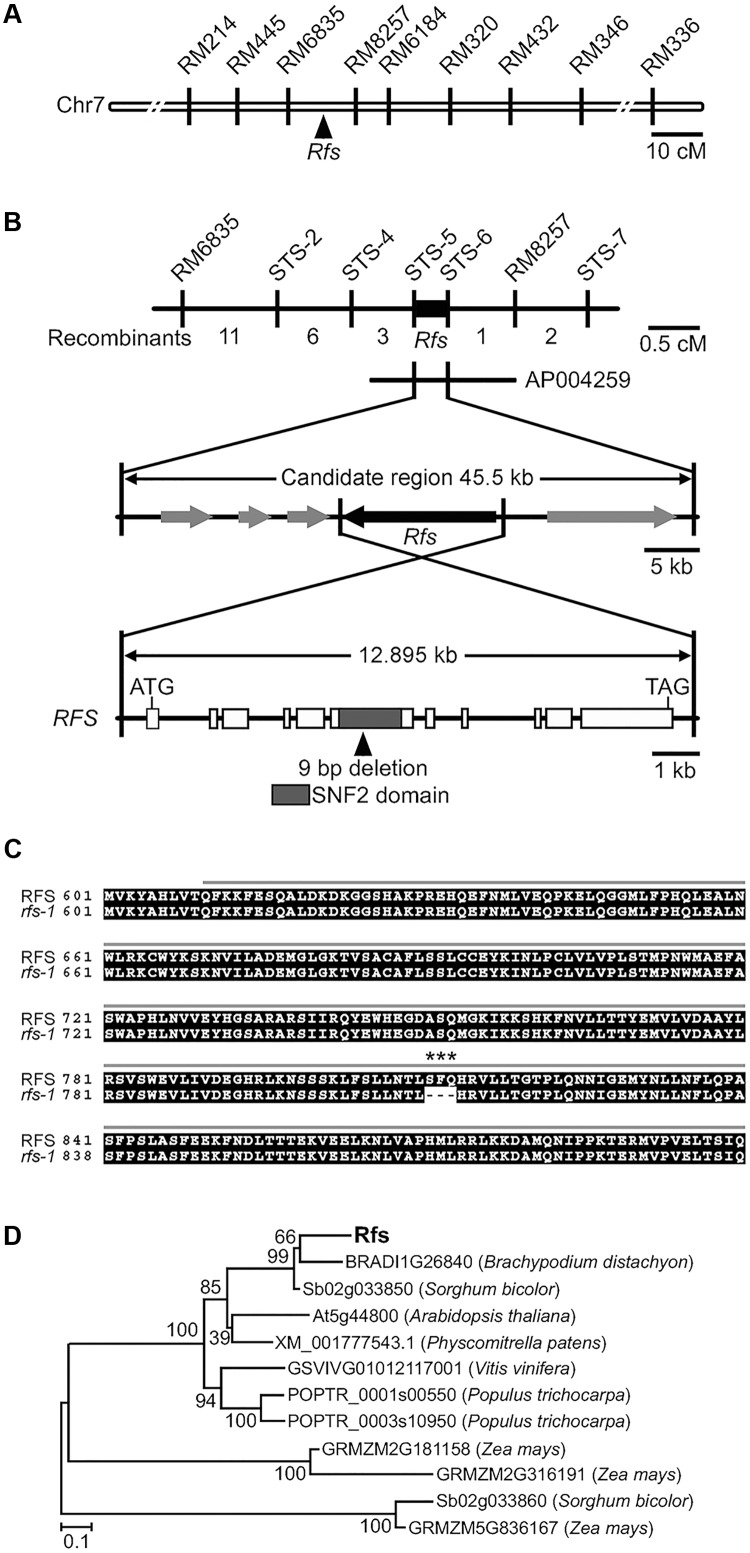
Map-based cloning of *RFS* and phylogenetic analysis of the RFS proteins in other plants. **(A)** Genetic mapping of the *RFS* gene using SSR and STS markers. **(B)** Physical mapping of the *RFS* gene. The genomic region contains five expressed genes (gray and black boxes). The genomic structure of *RFS*, comprising 11 exons and 10 introns, is indicated. A deletion mutation (SFQ 811–813) was found in exon 6 of *RFS* in the *rfs-1* mutant. **(C)** Nucleotide mutations result in deletion of the amino acids SFQ (asterisks) in the SNF2 family N-terminal domain (gray line). **(D)** The phylogenetic tree was constructed by the neighbor-joining method with MEGA (ver 5.1) program. Branch numbers represent percentage of bootstrap values in 1000 sampling replicates and the scale indicates branch length.

The *rfs* mutation is a single recessive allele whose locus has been mapped to an interval of 66 cM on chromosome 7^4^. To isolate the *RFS* gene, we mapped the *rfs-1* mutation to the long arm of chromosome 7 between the markers RM6835 and RM8257 (**Figure 4A**), based on the analysis of 591 F_2_ and 755 F_3_ plants from a cross between *rfs-1* and Milyang23 (a Tongil-type *indica*/*japonica* hybrid cultivar). *RFS* was then fine-mapped to a 45.5-kb region using both STS and SSR markers (**Figure 4B**).

This candidate region contains five putative ORFs; Os07g31420, Os07g31430, Os07g31440, Os07g31450, and Os07g31460, as annotated in the Rice Annotation Project Database^5^. We cloned cDNAs for these five genes by RT-PCR of total RNAs extracted from the young leaves of WT and *rfs-1*. Comparison of the sequences of the five ORFs between WT and *rfs-1* revealed a 9-bp deletion within the sixth exon of Os07g31450, which was annotated as encoding a putative CHR4/Mi-2-like chromatin remodeling factor (**Figure 4C**). *RFS* encodes a 2237-amino acid (aa) protein and has 11 exons and 10 introns. Phylogenetic analysis revealed that *O. sativa* RFS belongs to the same clade as *Brachypodium distachyon* RFS and *Sorghum bicolor* RFS (**Figure 4D**).

To demonstrate that the *rfs-1* phenotype is caused by a functional deficiency of the CHR4/Mi-2 chromatin remodeling factor protein, we performed genetic complementation using a T-DNA insertion mutant of Os07g31450. First, we searched for a T-DNA insertion mutant in the RiceGE (Rice Functional Genomic Express) database^6^. One T-DNA insertion mutant of Os07g31450 was obtained from the Crop Biotech Institute at Kyung Hee University (Jeong et al., 2006); this allele, 3D-02766, contains a T-DNA insertion in the 10th intron of Os07g31450 (Supplementary Figure S2A). Homozygous mutant plants were selected by PCR analysis, and the absence of Os07g31450 transcripts was confirmed by reverse transcription (RT)-PCR (Supplementary Figure S2D). Based on these results, we named the T-DNA insertion mutant line *rfs-2.* Consistent with the previous characterization of *rfs-1*, the *rfs-2* mutant had a rolled-leaf phenotype, reduced leaf width, and white variegated leaves (Supplementary Figures S2B,C). In transverse sections, the *rfs-2* leaves displayed defective vascular cells in large and small veins (Supplementary Figures S2E–G,I–K) and irregular bulliform cells (Supplementary Figures S2H,I) compared with the WT leaves. In addition, *rfs-2* mutants showed an accumulation of ROS (Supplementary Figure S4).

For the genetic complementation test, we crossed *rfs-1* with *rfs-2* mutants and analyzed the F_1_ and F_2_ plants. In the F_1_ generation, the plants showed a characteristic rolled-leaf finely striped *rfs* phenotype (Supplementary Figure S3A). We confirmed the heterozygous genotype of the F_1_ plants by PCR analysis (Supplementary Figure S3B). These observations show that the *rfs-1* and *rfs-2* alleles fail to complement each other, showing that they are alleles of the same gene and that mutations in Os07g31450 are responsible for the rolled-leaf, fine-striped phenotype.

### RFS Is Mostly Expressed in Developing Tissues in Rice

**FIGURE 5:**
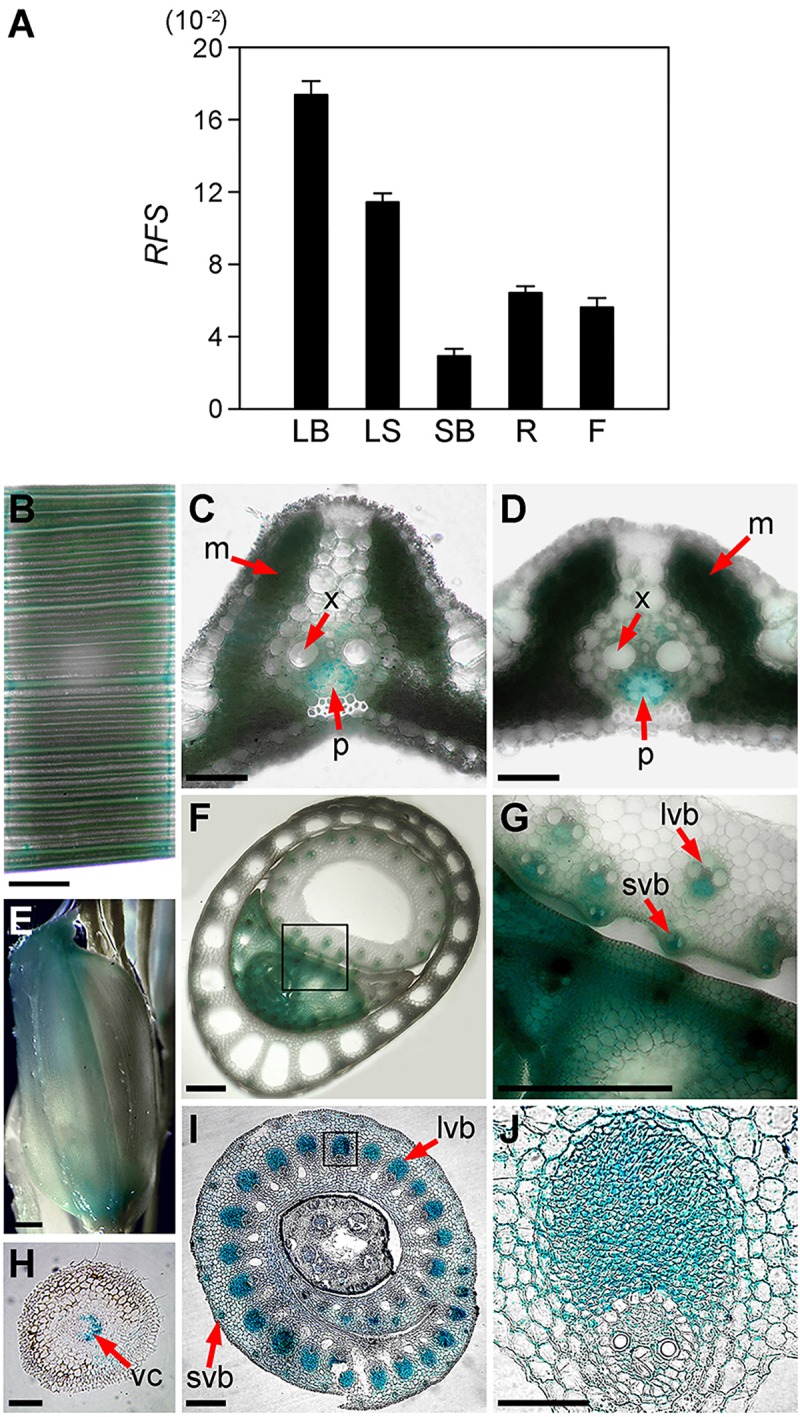
Expression profiles of *RFS*. **(A)** RT-qPCR analysis of *RFS* expression in various tissues. Total RNA was isolated from the leaf blade (LB), leaf sheath (LS), shoot base (SB), root (R), and flower (F) of WT. Relative expression levels of *RFS* were obtained by normalizing to the transcript levels of *OsUBQ5*. **(B–J)** Histochemical GUS analysis of the transgenic plants containing the *Pro_RFS_:GUS* construct. GUS activity was detected in the mesophyll cells (m) and leaf veins of the leaf blade **(B–D)**, especially in the phloem (p) but not in xylem (x) tissues of vascular bundle, longitudinal veins of a spikelet **(E)**, transverse section of leaf blade and node **(F,G)**, vascular cylinder (vc) of primary root **(H)**, and transverse section of large and small vascular bundle in basal node **(I,J)**. Scale bars: 1 mm **(B,E,H)**; 200 μm **(C,D)**; 500 μm **(F,G,I,J)**.

To investigate the expression pattern of *RFS*, we examined *RFS* transcript levels in various rice tissues by RT-qPCR analysis. The *RFS* transcripts were expressed abundantly in developing leaf blades and leaf sheaths, at low levels in shoot bases, and at moderate levels in roots and flowers (**Figure 5A**). These results showed that *RFS* is expressed in all plant tissues and most abundantly in developing leaf tissues.

To further characterize the *RFS* expression pattern, we employed a reporter gene fusion strategy. The 1,893-kb region upstream of the start codon was fused to the *GUS* reporter gene and we analyzed the transgenic plants containing the *Pro_RFS_:GUS* construct by histochemical GUS staining. In the leaves, GUS staining revealed that *RFS* was expressed mainly in the vascular tissues and mesophyll cells (**Figures 5B–D**). *RFS* expression was also detected in large and small vascular bundles of leaf sheaths and culms (**Figure 5G**), with especially high expression in the entire developing leaf while it was inside the leaf sheaths (**Figure 5F**). During the reproductive stage, *RFS* expression was observed in vascular strands of the whole spikelets with strong expression at both ends of the spikelets (**Figure 5E**). In roots, *RFS* expression was clearly detected in the central vascular cylinder (**Figure 5H**). Furthermore, *RFS* expression was observed in all differentiating vascular bundles in the central part (**Figures 5I,J**). These results showed that *RFS* was expressed in most rice vascular tissues and strongly expressed in developing leaf tissues, suggesting that *RFS* may play important roles in vascular development at the actively growing developmental stages.

### The Expression of ROS-Related Genes Is Altered in the *rfs-2* Mutants

**FIGURE 6:**
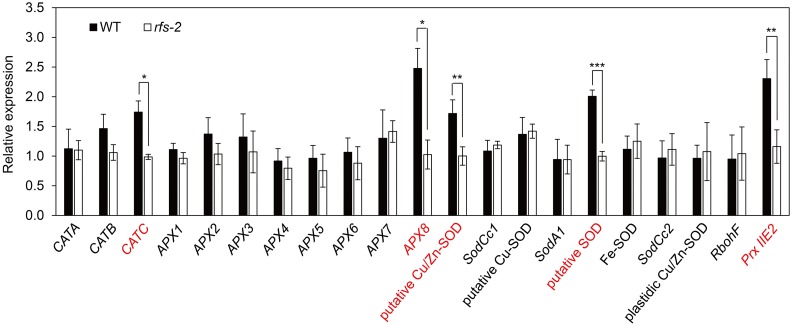
Altered expression of ROS-related genes in the WT and *rfs-2*. By RT-qPCR, the expression levels of 21 genes encoding the ROS scavengers were measured in the leaf tissues of 2-month-old WT and *rfs-2* (see Supplementary Figure S2C). The five genes whose expression was significantly decreased in the *rfs-2* leaves compared with in WT leaves are shown in red type. *UBQ5* was used as an internal control for RT-qPCR. *CATA;* catalase A, *CATB;* catalase B, *CATC*, catalase C; *APX1*, ascorbate peroxidase 1; *APX2*, ascorbate peroxidase 2; *APX3*, ascorbate peroxidase 3; *APX4*, ascorbate peroxidase 4; *APX5*, ascorbate peroxidase 5; *APX6*, ascorbate peroxidase 6; *APX7*, ascorbate peroxidase 7; *APX8*, ascorbate peroxidase 8; putative Cu/Zn-SOD, a putative copper/zinc superoxide dismutase (SOD); *SodCc1*, Cu/Zn-SOD 1; putative Cu-SOD, a putative chaperone for copper SOD; *SodA1*, putative SOD; *SodCc2*, Cu/Zn-SOD 2; *RbohF*, NADPH oxidase RbohF; *Prx IIE2*, peroxiredoxin IIE2. Bars represent ± SD from three independent experiments. Asterisks indicate statistically significant difference compared to WT as determined by Student’s *t*-test (^∗^*P* < 0.05, ^∗∗^*P* < 0.01, ^∗∗∗^*P* < 0.005).

Superoxide dismutase, APX, CAT, and PrxR are major ROS-scavenging enzymes that help maintain ROS homeostasis (Mittler et al., 2004). We used RT-qPCR to measure the expression levels of 21 genes encoding SODs, APXs, CATs, PrxR, and NADH oxidase (Fang et al., 2015) in the leaf blades of 2-month-old WT and *rfs-2* plants (**Figure 6**). The transcript levels of five ROS-related genes including *CATC, APX8*, a putative copper/zinc superoxide dismutase (*Cu/Zn-SOD*) gene, a putative *SOD* gene, and *Prx IIE2* (peroxiredoxin IIE2) were dramatically decreased in the *rfs-2* mutants. However, expression of 15 other ROS-related genes showed no significant difference in the *rfs-2* mutants. These results suggested that RFS may influence the expression of several ROS-related genes, and reduction of ROS-related gene expression in the *rfs-2* mutants contributes to their failure to cope with ROS toxicity.

### RFS Affects Histone Modifications at ROS-Related Genes

**FIGURE 7:**
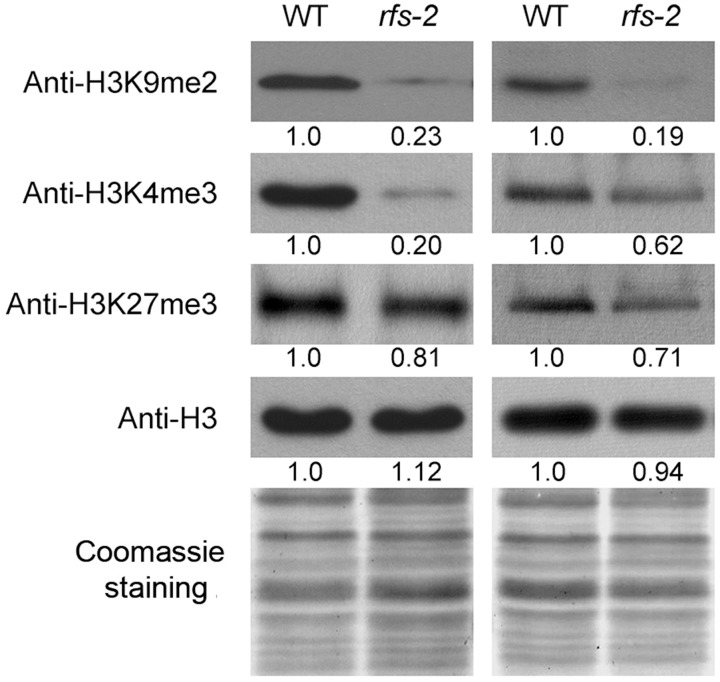
Accumulation of modified histone proteins in the WT and *rfs-2*. Enriched histone fractions were isolated from 2-week-old WT and *rfs-2* seedlings grown in the growth chamber. Western blots were performed by using antibodies against dimethylated H3K9, trimethylated H3K4, and trimethylated H3K27. The blotted membrane was stained with Coomassie Brilliant Blue reagent to show equal loading of each lane. The levels of each protein are shown relative to those of WT, which is set as 1.

Rolled Fine Striped is annotated as a CHR4/Mi-2 chromatin remodeling factor, which can potentially affect histone methylation. To test whether histone protein modifications are altered in the *rfs* mutants, we isolated histones from the leaves of 2-week-old WT and *rfs-2* and analyzed them by western blots using specific antibodies against H3K4me3, H3K9me2, and H3K27me3. Indeed, reduced levels of H3K4m3 and H3K27me3 were observed in the *rfs-2* mutants (**Figure 7**), consistent with a previous report (Hu et al., 2012). Moreover, we found that the *rfs-2* mutants had lower levels of H3K9 dimethylation (H3K9me2) compared with WT.

**FIGURE 8:**
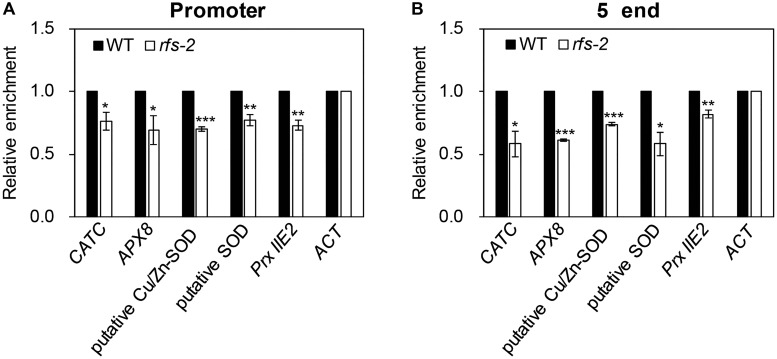
OsRFS modulates H3K4me3 levels on ROS-related genes. Chromatin immunoprecipitation (ChIP) analysis of H3K4me3 on the selected ROS-related genes in the leaves of 2-month-old WT and *rfs-2* (see Supplementary Figure S4). Enrichment of H3K4me3 on the promoter **(A)** and the 5′ end region **(B)** of the selected ROS-related genes was measured by ChIP followed by qPCR. *CATC*, *APX8*, putative Cu/Zn-SOD, putative SOD, *Prx IIE2*. *OsActin7* (*ACT*) was used for the normalization of the qPCR analysis. Means and standard deviations were obtained from three biological replicates. Asterisks indicate statistically significant difference compared to WT as determined by Student’s *t*-test (^∗^*P* < 0.05, ^∗∗^*P* < 0.01, ^∗∗∗^*P* < 0.005).

Histone modification can affect gene transcription by determining the accessibility of a genomic region to transcription factors and/or transcription machinery (Pfluger and Wagner, 2007). For example, trimethylation of histone lysine 4 (H3K4me3) is generally correlated with gene activation (Li et al., 2008; Zhang et al., 2009). This evidence prompted us to determine whether RFS was required for depositing H3K4 trimethylation on ROS-related genes. To this end, the leaf blades of 2-month-old WT and *rfs-2* mutants were collected for ChIP analysis. We performed ChIP assays for H3K4me3 on *CATC*, *APX8*, a putative *Cu/Zn-SOD* gene, a putative *SOD* gene, and *Prx IIE2*. H3K4me3 is limited to the transcribed regions of genes, with a slight bias toward the 5′ end and the proximal promoter (Alvarez-Venegas, 2005; Pfluger and Wagner, 2007). Therefore, for qPCR to analyze the ChIP results, we used two primer sets, one corresponding to the promoter and the other to the 5′ end region. The levels of H3K4me3 were decreased in the five selected genes in the *rfs-2* mutants (**Figure 8**). To further investigate whether the modification of H3K4me3 specifically occurs in the five representative genes, we measured the H3K4me3 levels in *CATA*, *APX7*, and *Fe-SOD* genes, which encode ROS-scavenging enzymes. This revealed that the relative enrichment of H3K4me3 on these three genes was not significantly altered in *rfs-2* mutant compared with WT (Supplementary Figure S5). Taken together, these results showed that RFS affects modification of histone proteins that directly bind to ROS-related genes to maintain ROS homeostasis.

### RFS Is Involved in Multiple Developmental Processes

Rolled Fine Striped affects multiple developmental processes, including chloroplast development, as described in a previous report, (**Figure 1** and Supplementary Figure S1; Zhao et al., 2012). Pollen development was defective in the *rfs-2* mutants compared with WT (Supplementary Figures S6A,B). In panicles, the *rfs-2* mutants had many sterile seeds (Supplementary Figure S6C; Hu et al., 2012). In seed, the outer glume was short and the seeds were narrow (Supplementary Figures S6D–F). In comparison with the WT, the primary roots and lateral roots were shorter and the mutants had fewer adventitious roots (Supplementary Figures S6G–I; Wang et al., 2016). Finally, the *rfs-1* mutants grown in the paddy field showed delayed heading, compared with WT (Supplementary Figure S6J). Therefore, *RFS* affects many aspects of plant development, consistent with its widespread expression throughout the plant (**Figure 5**).

## Discussion

Mutant alleles of *CHR729* encoding a CHD3/Mi-2 protein have been isolated by various approaches. Molecular genetic studies revealed that CHR729 affects multiple aspects of plant development, including chloroplast formation, seedling development, and root elongation (Hu et al., 2012; Zhao et al., 2012; Ma et al., 2015; Wang et al., 2016). In this study, a new *rfs* mutant allele that contains a 9-bp deletion in *CHR729* was isolated from a gamma ray-treated rice population. The *rfs* mutants had fewer large and small veins and exhibited a rolled and fine-striped phenotype on the adaxial side of leaf blades (**Figures 1**, **4**). This phenotype strongly suggested that RFS is involved in development of the vasculature during leaf development. In the *rfs* leaves, the white stripes developed only on the adaxial side but the density and distribution of mesophyll cells were altered on both the abaxial and adaxial sides (**Figures 1**, **2**). In *Arabidopsis*, the reticulated mutants have mesophyll-specific defects and studies of these mutants have identified genes that are responsible for the development of mesophyll cells (Lundquist et al., 2014). For instance, the number of mesophyll cells is reduced in the *reticulata* (*re*) and *reticulata-related 3* (*rer3*) mutants. Accumulation of H_2_O_2_ in the *re* and *rer3* mutants attenuates mesophyll differentiation, resulting in a reticulated-leaf phenotype (Berná et al., 1999; Pérez-Pérez et al., 2013). Moreover, in *Arabidopsis*, functional deficiency of *NADPH-Thioredoxin Reductase* (*NTRC*) caused growth retardation with small and irregular mesophyll cells. The rice NTRC enzyme reduces H_2_O_2_ using NADPH as the source of reducing power (Pérez-Ruiz et al., 2006). Thus, plant species have evolved diverse mechanisms to maintain ROS homeostasis to prevent damage to the mesophyll development. The observation that the *rfs* mutants not only impaired mesophyll cell development but also accumulated ROS (**Figure 3**) indicated that RFS is closely related to mesophyll development through ROS scavenging. Indeed, null mutation of *RFS* led to a decrease in the expression of ROS-scavenging genes, resulting in high accumulation of ROS in *rfs* mutant leaves (**Figures 3**, **6**). Therefore, RFS is required for the expression of specific genes for maintaining ROS homeostasis. Furthermore, loss-of-function of RFS reduces the trimethylation of histone H3K4 in the ROS-related genes. This study revealed the role of RFS/CHR729 in epigenetic regulation of the expression of ROS-related genes.

### Altered Expression of ROS-Scavenging Genes Causes Chlorosis and Cell Death in the *rfs* Leaves

We found that more ROS accumulated in *rfs* mutant leaves compared to the WT, resulting in chlorotic phenotypes (**Figure 3** and Supplementary Figure S4). Furthermore, comparison of chloroplasts between WT and the white sectors of *rfs-1* showed that the photosynthetic organelles such as grana and thylakoids disappeared and plastoglobules, which are often coupled to the thylakoid membranes, formed larger clusters in the *rfs-1* mutants (Supplementary Figure S1). Plastoglobules have important roles in lipid biosynthesis and storage subcompartments of thylakoid membranes. Their number and size increase when plant cells are exposed to oxidative stress and during senescence (Austin et al., 2006). Therefore, this observation suggested that excessive accumulation of ROS in the *rfs* leaves destroys the lamellar structure and develops more plastoglobules and small vacuole-like structures. ROS are produced by metabolic activity such as photosynthesis and photorespiration, and by external stimuli such as biotic/abiotic stress; excess ROS must be detoxified to alleviate oxidative stress (Dat et al., 2000; Polle, 2001; Mittler et al., 2004). Higher plants have developed ROS-scavenging mechanisms to maintain a balance between ROS production and destruction (Miller et al., 2007). These mechanisms include several ROS-scavenging enzymes such as SOD, APX, glutathione peroxidase, CAT, and PrxR (Noctor and Foyer, 1998; Apel and Hirt, 2004; Mittler et al., 2004). APX and CAT are essential antioxidant enzymes that function in converting H_2_O_2_ into H_2_O and O_2_ (Asada, 1999). The rice genome contains eight genes encoding APX enzymes with different localizations: OsAPX1 and OsAPX2 localize in the cytosol, OsAPX3 and OsAPX4 in peroxisomes, OsAPX6 in mitochondria, and OsAPX5, OsAPX7, and OsAPX8 in chloroplasts (Teixeira et al., 2006). Overexpression of thylakoid membrane-bound OsAPX8 improved tolerance to bacterial pathogens and *OsAPX8* transcript levels increased in response to exogenously applied NaCl and abscisic acid (Hong et al., 2007; Jiang et al., 2016). The rice genome contains three *CAT* isoenzyme genes, *OsCATA*, *OsCATB*, and *OsCATC*. *OsCATC* is expressed in the leaf blade and abscisic acid suppresses its expression (Iwamoto et al., 2000; Agrawal et al., 2001). SOD catalyzes the dismutation of O_2_^-^ to H_2_O_2_ and O_2_. Based on the metal co-factor used, SODs can be classified as copper/zinc SOD (Cu/Zn-SOD), manganese SOD (Mn-SOD), and iron SOD (Fe-SOD) (Ueda et al., 2013; Yin et al., 2014). Cu/Zn-SOD and Fe-SOD are located in the chloroplast (Donahue et al., 1997; Ueda et al., 2013).

Based on the overaccumulation of ROS revealed by histochemical staining, we speculated that expression of ROS-related genes might be altered in the *rfs-2* mutant. Indeed, RT-qPCR analysis showed that transcript levels of five ROS-related genes significantly decreased in *rfs-2* mutants compared to the WT (**Figure 6**). Furthermore, OsAPX8, OsCATC, and Cu/Zn-SOD are mainly found in chloroplasts and expressed in the leaf blade, consistent with *RFS* expression (**Figure 5**; Zhao et al., 2012). These findings provided evidence that RFS might be tightly connected to the regulation of ROS-related genes. Taken together, our results show that null mutation of *RFS* leads to a reduction in expression of ROS-related genes, overaccumulation of endogenous ROS, and finally leaf chlorosis and cell death.

### Epigenetic Regulation by RFS

Chromatin remodelers affect histone modifications, thus epigenetically regulating gene expression. Loss of RFS causes a decrease in the trimethylation of histone H3K4 and H3K27 (**Figure 7**), consistent with a previous report that loss of *CHR729* decreases H3K4me3 and H3K27me3 (Hu et al., 2012). This study further demonstrates that dimethylation of histone H3K9 is reduced in the *rfs-2* mutant (**Figure 7**). The PHD finger of CHD3 proteins can bind methylated histones such as H3K4me3 and H3K27me3 (Sanchez and Zhou, 2011; Hu et al., 2012). For instance, the HP1 chromodomain binds to H3K9me2/me3 in animal cells (Vermaak and Malik, 2009). However, *Arabidopsis* LHP1, which has high sequence similarity to HP1, recognizes H3K27me3 deposited by the POLYCOMB REPRESSIVE COMPLEX 2 (PRC2), instead of H3K9me2/me3 (Turck et al., 2007). No alteration of H3K27me3 was observed in the *lhp1* mutant, but loss of RFS significantly decreased H3K9me2, H3K4me3, and H3K27me3 (**Figure 7**; Hu et al., 2012). Moreover, *Arabidopsis* PKL indirectly promotes H3K27me3 by promoting expression of the PRC2 complex. Therefore, PKL-dependent genes were enriched for H3K27me3, a repressive epigenetic mark (Zhang X. et al., 2007; Zhang et al., 2012). These results indicate that RFS functions in epigenetic regulation by depositing methylation marks on histone proteins.

Histones modified by methylation play important roles in the epigenetic regulation of gene expression. Among those modifications, H3K4me3 is largely known as an activation mark, since it is mostly found in actively transcribed genes (Alvarez-Venegas, 2005; Pfluger and Wagner, 2007). In *Arabidopsis*, trimethylation of H3K4 is established by SET DOMAIN GROUP 2 (SDG2), a histone methyltransferase (HMT) containing the SET lysine transferase catalytic domain (Ng et al., 2007; Guo et al., 2010; Zhu et al., 2011). Furthermore, H3K4 trimethylation marks are found in loci that are which transcriptionally regulated by the ARABIDOPSIS HOMOLOG OF TRITHORAX1 (ATX1)/AtCOMPASS complex. ATX1/AtCOMPASS is required for assembling a pre-initiation complex and allowing RNA Polymerase II (Pol II) to enter the initiation phase of transcription. However, these processes are independent of H3K4me3 marks on the ATX1-regulated genes. Instead, the trimethylation of H3K4me3 can help to facilitate the transition of Pol II to the elongation phase of transcription (Jiang et al., 2011; Ding et al., 2012; Fromm and Avramova, 2014). In rice, previous genome-wide analysis indicated that loss of H3K4me3 is correlated with gene down-regulation in *chr729* mutants (Hu et al., 2012). In this study, we found that suppression of H3K4me3 by functional deficiency of RFS led to a decrease in expression of five ROS-related genes (**Figure 8**). The possibility that RFS directly deposits H3K4me3 on ROS-related genes could be excluded, because RFS lacks the SET domain conferring HMT catalytic activity (Flaus et al., 2006; Hu et al., 2014). Therefore, these findings indicate that RFS may recruit HMTs to establish the trimethylation of H3K4 on ROS-related genes. One model suggested that in *Arabidopsis*, PKL could interact with one or more PRC2 complexes to deposit H3K27me3 marks on PKL-regulated genes (Zhang et al., 2008). The animal CHD3/Mi-2 proteins form the Nucleosome Remodeling Deacetylase complex, which is composed of histone deacetylases and has histone deacetylation activity on target genes (Denslow and Wade, 2007). The HMTs that contribute to the trimethylation of H3K4 by directly interacting with RFS remain to be discovered.

## Author Contributions

S-HC, H-JK, KK, and N-CP designed the research. S-HC performed the physiological, biochemical, and genetic experiments. C-HL characterized the mutant and performed map-based cloning. KK, EG, and YY designed and performed the ROS analyses, ChIP assay, and gene expression analysis. S-HC, and KK analyzed the data. S-HC, KK, and N-CP wrote the article.

## Conflict of Interest Statement

The authors declare that the research was conducted in the absence of any commercial or financial relationships that could be construed as a potential conflict of interest.

## Footnotes

1 http://www.gramene.org/markers/

2 http://www.genome.arizona.edu/fpc/rice/

3 http://rsb.info.nih.gov/ij/docs/menus/analyze.html#gels

4 https://shigen.nig.ac.jp/rice/oryzabase/gene/detail/816

5 http://rice.plantbiology.msu.edu

6 http://signal.salk.edu/cgi-bin/RiceGE

## Abbreviations

APX: ascorbate peroxidase
CAT: catalase
CHD: chromodomain, helicase/ATPase, DNA-binding
ChIP: chromatin immunoprecipitation
DAB: 3,3′-diaminobenzidine
NBT: nitroblue tetrazolium
PrxR: peroxiredoxin
*RFS*: *Rolled Fine Striped*
ROS: reactive oxygen species
RT-qPCR: reverse transcription-quantitative polymerase chain reaction
SNF2: sucrose non-fermenting
SOD: superoxide dismutase
SOSG: singlet oxygen sensor green
T-DNA: transfer-DNA
WT: wild type.
