# Supplementary material for: The Rice Rolled Fine Striped (RFS) CHD3/Mi-2 Chromatin Remodeling Factor Epigenetically Regulates Genes Involved in Oxidative Stress Responses During Leaf Development
Source: Front Plant Sci. 2018 Mar 20;9:364. doi: 10.3389/fpls.2018.00364 (PMC5870552; doi:10.3389/fpls.2018.00364)
Supplement: Supplementary file 1 [file Table_1.DOCX]

**TABLE S1**. Primers used in this study

| A. Physical mapping markers | | | | |
| --- | --- | --- | --- | --- |
| Locus | Contig | Location | Forward primer (5’→3’) | Reverse primer (5’→3’) |
| RM6835 | AY023510 |  | TTCTGCTCCACGTGTTCTTG | TAACCCATAGTCCCGTACGC |
| STS-1 | AY023510 | 40 kb | AAACCACGCGATCATCAGAT | ACCGAGAGGCTTCTCCGTTT |
| STS-2 | AY023510 | 49.4 kb | GATGACACCGATGACTTTC | GATGACACCGATGACTTTC |
| STS-3 | AP003956 | 72.5 kb | TGTTACTACGATTCTGGACAG | CATTCATTCATAAGTCGTTCC |
| STS-4 | AP003956 | 9.5 kb | CAACAACTTGTCAGGTGGGTAC | GGGTGTACATATGATGATTCC |
| STS-5 | AP004259 | 15.8 kb | TAGGACTGAGGGTGTACATATG | ATAAATCTGGACAGGCTCTATG |
| STS-6 | AP004259 | 42.4 kb | CCTCTGCTCTGATCGATTTC | TCCTCATCCGACTTCTCTTG |
| RM8257 | AP004259 | 55 kb | GTGACACTGGAAAGGATTTG | CCCACATATGATTGACCTTC |
| STS-7 | AF344145 | 60.1 kb | AACGACCACTCATTTAATAAGC | TTGTAGACCGTGTCAAGTTTG |
| RM320 | AF344145 |  | CAACGTGATCGAGGATAGATC | GGATTTGCTTACCACAGCTC |
| B. Primers for proRFS:GUS construction | | | | |
| Product | | | Forward primer (5'→3') | Reverse primer (5'→3') |
| RFS promoter | | | CGGAAAATAGGCAACCCTTA | CTTTTAGCCCACGTGGAAGT |
| C. Primers for RT-qPCR | | | | |
| Product | | | Forward primer (5'→3') | Reverse primer (5'→3') |
| RFS | | | CGTCTCTCTCTCCCCAAGGA | CAGGGAGGAAGCTTGCTGAA |
| CATC | | | GATGAGTCGTTGGGGGTGAA | CACATGCTTGGCTTCACGTT |
| CATB | | | TGGATCTCCTACTGGTCGCA | TCAGGTTGAGACGTGAAGCC |
| CATC | | | CGAAGCCGAGCATGTAAGGA | GGCTGAGGCCTTCCGTG |
| APX1 | | | TACAAGGAGGCCCACCTCAA | TAAGCATCAGCGAACCCCAG |
| APX2 | | | ACAAAGCCCTCATGGCTGAC | TCGGCGTAATCCGCAAAGAA |
| APX3 | | | AGCACTCTCAGGCGGCC | AGGCACCATCAAATCCTGATCT |
| APX4 | | | CGGCCCTGCATCTACGAA | CAGCTACCCCGACAGCACTC |
| APX5 | | | CCGGTGGCCCCAAGAT | GGTGGGCATTCTTCACCATC |
| APX6 | | | CCCCAAGATCCCCATGATCTA | CCTCTGGCGGGCATTG |
| APX7 | | | TTCACGTTGGACGGTTAATGC | TTTCTGTAAAAGTGGTTGGCCA |
| APX8 | | | ATCATCGCCAGCGGATGA | GCAGCGACGAAGGGCTC |
| putative Cu/Zn-SOD | | | CAGATTTCACTAAGCGGGCC | CTTCCTAGGTCATCAGAATCAGCA |
| SodCc1 | | | TGCGCATGGTTTTTGGTG | CATAGTTCATTGGGCGCCA |
| putative Cu-SOD | | | AGATCAGATCCTGGCATTGCA | TTCTTGTAGTTCTCGCCAACCC |
| SodA1 | | | CCGCCATCGTGCACCT | TCGAATGATTGACATGGCCTC |
| putative SOD | | | CGGAGCCAAGTAAGCTACGG | TGTTACAAGTCCATCATCCCCA |
| Fe-SOD | | | CGACGCCGAGGAATTTCTAG | AGGTGGTGTAAGTGTCTCTCATGC |
| SodCc2 | | | ATTCCATGTGCACGCGC | GGATTGAAGTGTGGTCCAGTTG |
| plastitic Cu/Zn-SOD | | | TGTGACGGGACTTACTCCTGG | CACCCATTCGTAGTATCGCCA |
| RbohF | | | TCCAATCAGTGTAGCGTGGC | TTTGAATGACCGGCGCTTTG |
| Prx IIE2 | | | GCGCTCTGAAGATGGAATCC | TCACAACGACGACGCTACTCA |
| OsUBQ5 | | | ACCACTTCGACCGCCACTACT | ACGCCTAAGCCTGCTGGTT |
| D. Primers for ChIP assay | | | | |
| CATC-promoter | | | CGGGTCAATGTGAACAGCATC | CGACGGCCAATGGGAATTTT |
| CATC-5`end | | | TCGTACGTAACGTGACCGAG | AGAGCAGAGCACGTTCGTTA |
| APX8-promoter | | | ACGAGGGAGAGAAAACCCAAA | ACCTTCGAGATGTCGCTTGA |
| APX8-5`end | | | CCCCATCATGGTACGGAACG | CATATGGATGCGAGAGCTGAGA |
| putative Cu/Zn-SOD-promoter | | | CCACATGAAGCAATTCAATTCTAGG | GCCAGGGACGGAATTTGTCT |
| putative Cu/Zn-SOD-5`end | | | CCATGGCTTCCACATCCACT | AAGGCAACGAACCGGTAGAG |
| putative SOD-promoter | | | ATAATGTGGCGGCCCACTAAG | TAATGTGGGCTTTGGGCTTCT |
| putative SOD-5`end | | | CGATGATGAAACCGCTTCCG | TCATCAGTGCCTGACGACTC |
| Prx IIE2-promoter | | | TCATAGGCCCAACCCGAATC | CCGAAACAGGATTGTTCGGC |
| Prx IIE2-5`end | | | TATTTCCTATCTCTCGCGTCG | TGCTTCTCTGTTGGGATTTGGT |
| CATC-promoter | | | ACCTCTTCTGCCGGATGTATG | CTCAAACGCAAAGGGGTCAC |
| CATC-5`end | | | TCGTACGTAACGTGACCGAG | AGAGCAGAGCACGTTCGTTA |
| APX7-promoter | | | CAACCGTGACGTGTCATCCTA | GAGAACGGTGACTGGTGCAG |
| APX7-5`end | | | AAAAACCCCCTCGAAACCCAAG | AAAAGTCCAGTCCACCACCACG |
| Fe-SOD-promoter | | | CGAGCAGAAAAGGGGACGAT | CGGACGAACAAGTTAACCGA |
| Fe-SOD-5`end | | | GGGATAGGAAGGGGAAGGGA | AAAAACCTGCAATGCCCTCG |
| OsActin7 | | | AGCACCACGAACCTTGACCAT | TGCGTCAGGAATTCAGAACCA |
| E. Primers for T-DNA verification in *rfs-2* mutant | | | | |
| Product | | | Forward primer (5'→3') | |
| *rfs-2*-LP | | | AGACTGTCGTTCCCATTTGC | |
| *rfs-2*-RP | | | ATGGAAATCTGAGGTGGCAC | |
| T-DNA BP | | | TTGGGGTTTCTACAGGACGTAAC | |
