# Supplementary material for: The Rice Rolled Fine Striped (RFS) CHD3/Mi-2 Chromatin Remodeling Factor Epigenetically Regulates Genes Involved in Oxidative Stress Responses During Leaf Development
Source: Front Plant Sci. 2018 Mar 20;9:364. doi: 10.3389/fpls.2018.00364 (PMC5870552; doi:10.3389/fpls.2018.00364)
Supplement: Supplementary file 2 [file Image_1.PDF]

Supplemental Data – Cho SH et al.

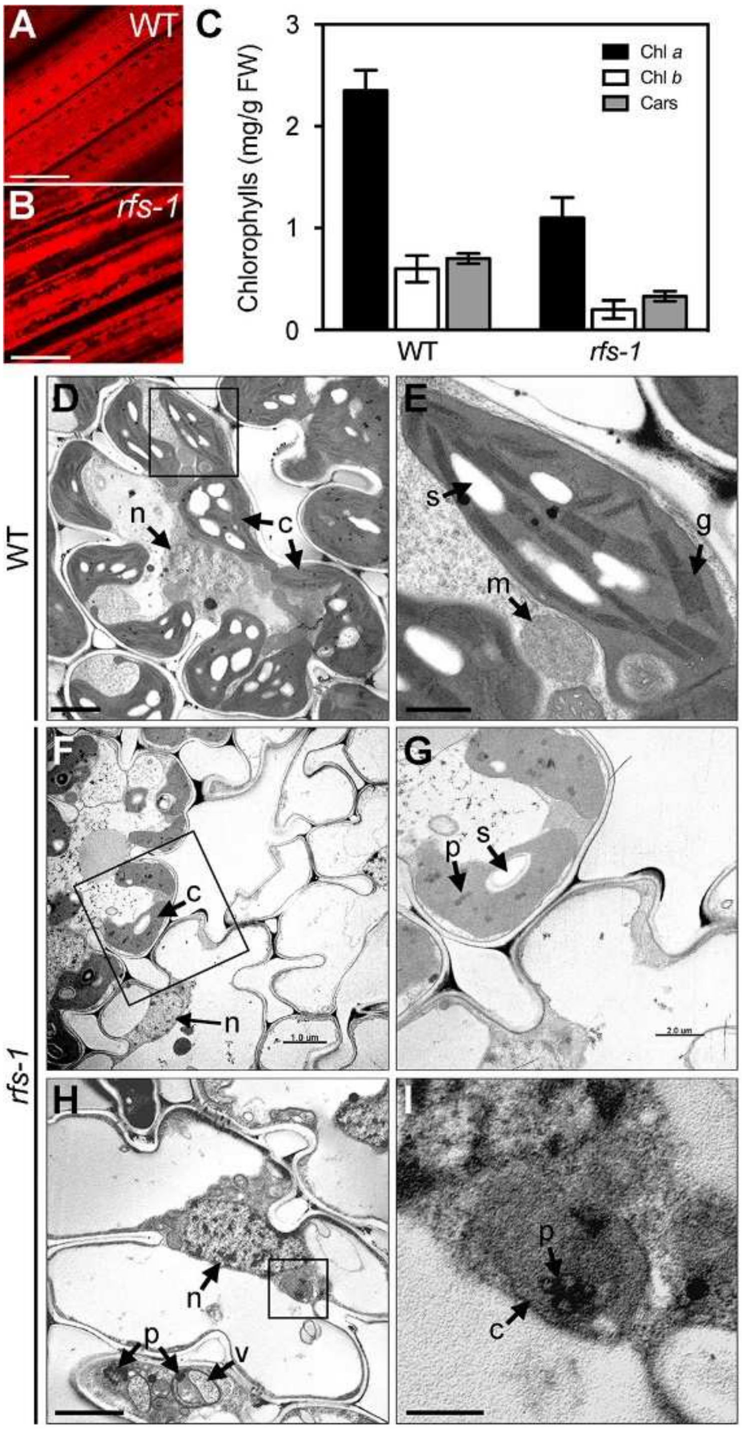

FIGURE S1. The *rfs-1* mutants showed impaired development of chloroplasts.

The leaf blade tissues of two-month-old WT and *rfs-1* grown in the paddy field were used. (**A, B**) Confocal micrograph of the WT and *rfs-1* leaves. (**C**) Chlorophyll and carotenoid contents of the WT and *rfs-1* leaves. Chl a, chlorophyll a; Chl b, chlorophyll b, Cars, carotenoids. (**D, E**) Transmission electron micrograph of green sector of a WT leaf. chloroplast (c); nucleus (n). (**E**) Enlarged of (**D**) in WT leaf. starch granule (s); mitochondria (m); grana (g). (**F, G**) Transmission electron micrograph of green sector of a *rfs-1* leaf. chloroplast (c); nucleus (n); plastoglobule (p). (**G**) Enlarged of (**F**) in *rfs-1* leaf. starch granule (s). (**H, I**) Transmission electron micrograph of white sector of a *rfs-1* leaf. nucleus (n); plastoglobule (p); vacuole-like (v). (**I**) Enlarged of (H) in *rfs-1* leaf. chloroplast (c); plastoglobule (p). Scale bars: 100 μm (A, B); 2 μm (D, F, H); 0.5 μm (E, G, I).

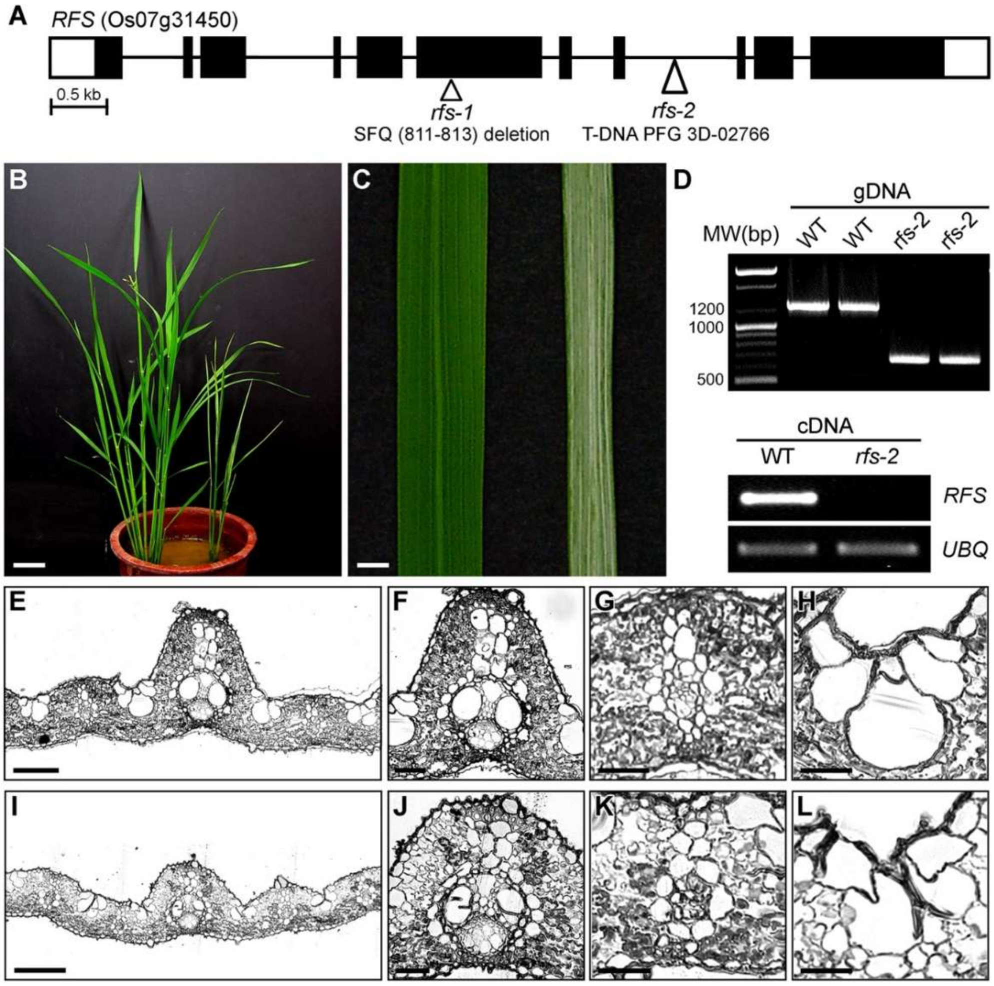

FIGURE S2. Analysis of the *rfs-2* null mutant line.

(A) Structure of the *RFS* gene and T-DNA insertion site. Exons are represented by black boxes. **(B)** Phenotypes of two-month-old WT (WT; left) and *rfs-2* (right). **(C)** Rolled and white variegated leaf of the *rfs-2* mutant. **(D)** T-DNA insertion in the *rfs-2* mutant was used as a PCR-based marker to distinguish the WT and *rfs-2*. RT-PCR analysis of the *RFS* transcripts in two-week-old WT and *rfs-2* grown in paddy field. gDNA, genomic DNA; MW, molecular weight; bp, base pair; cDNA, complementary DNA; UBQ, *ubiquitin 5*. **(E, I)** Transverse section of WT and *rfs-2* leaves. **(F, J)** Transverse section of WT and *rfs-2* large vein. **(G, K)** Transverse section of WT and *rfs-2* small vein. **(H, L)** Transverse section of WT and *rfs-2* bulliform cells. Scale bars: 5 cm **(B)**; 5 mm **(C)**; 100 μm **(E, I)**; 50 μm **(F, G, H, J, K, L)**.

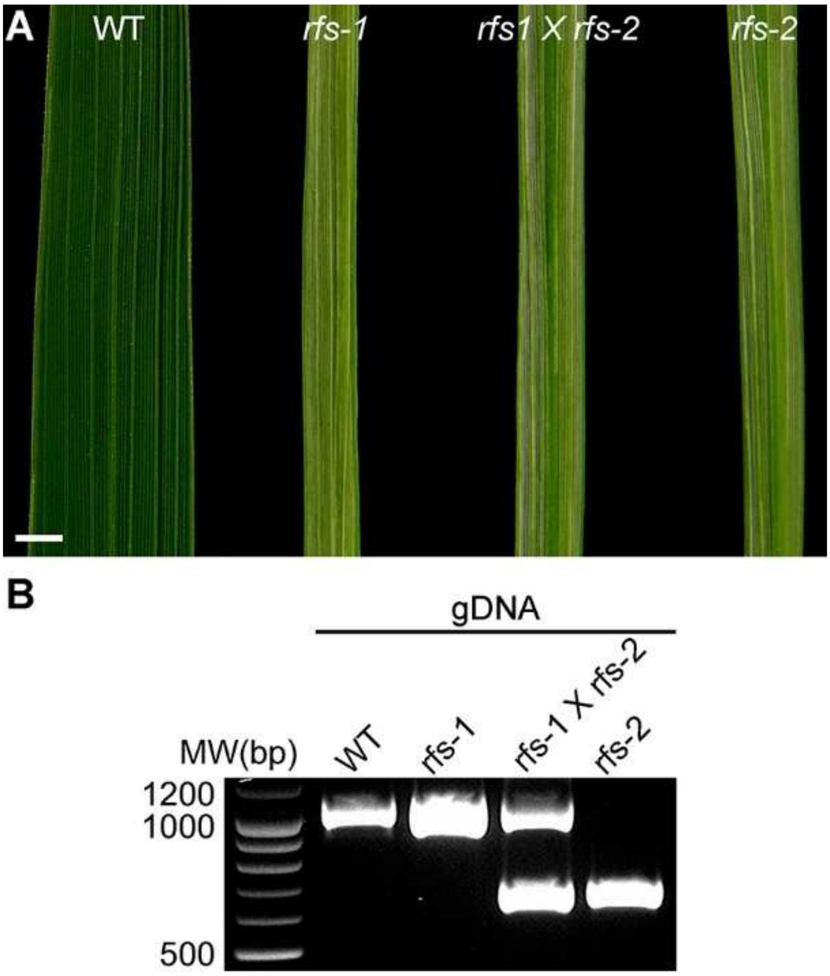

FIGURE S3. Genetic complementation of the *rfs-1* and T-DNA insertion *rfs-2* mutants.
(A) Phenotype of *rfs-1* (left), *rfs-1* X *rfs-2* (middle), and *rfs-2* (right). **(B)** T-DNA insertion in the *rfs-2* mutant was used as a PCR-based marker to distinguish the three plants. Scale bar: 5 mm **(A)**. gDNA, genomic DNA; MW, molecular weight; bp, base pair.

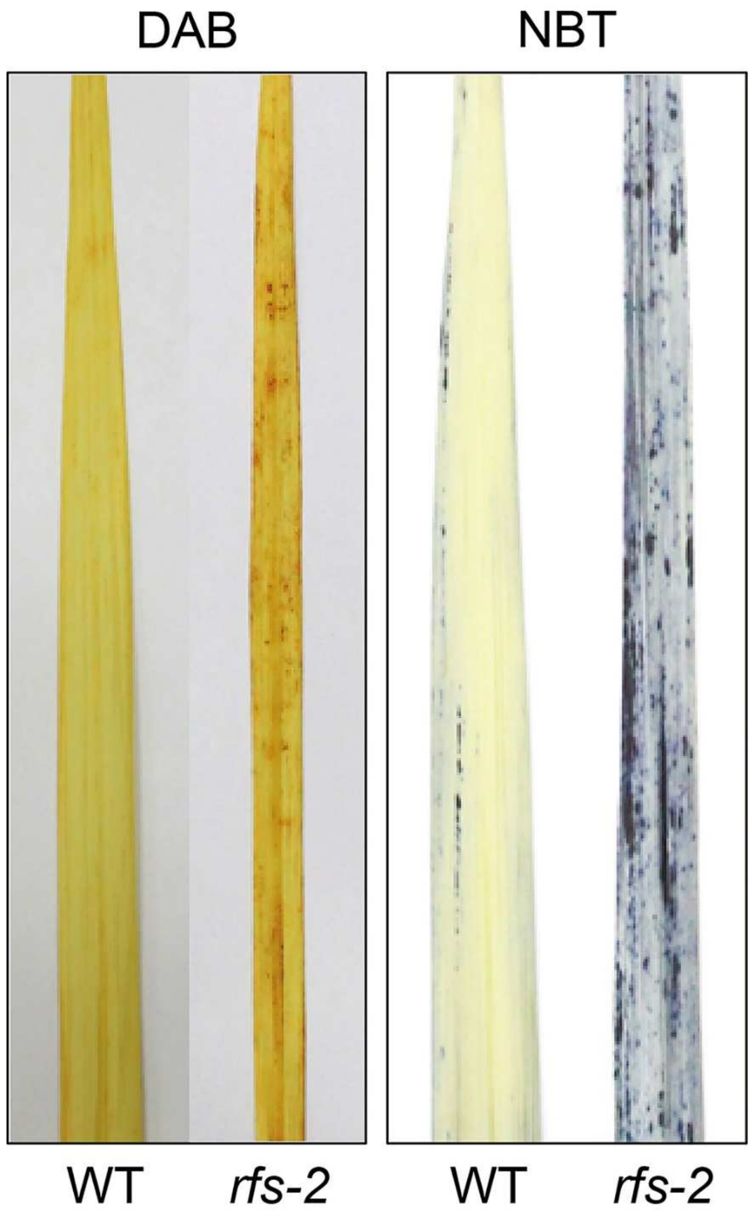

FIGURE S4. ROS accumulation in the *rfs-2* mutant.

DAB staining for hydrogen peroxide (H_2O_2) (dark brown) and NBT staining for superoxide radical (O_2^-) (blue) in leaves of two-month-old WT and *rfs-2* grown in paddy field. DAB, 3,3'-diaminobenzidine; NBT, nitroblue tetrazolium.

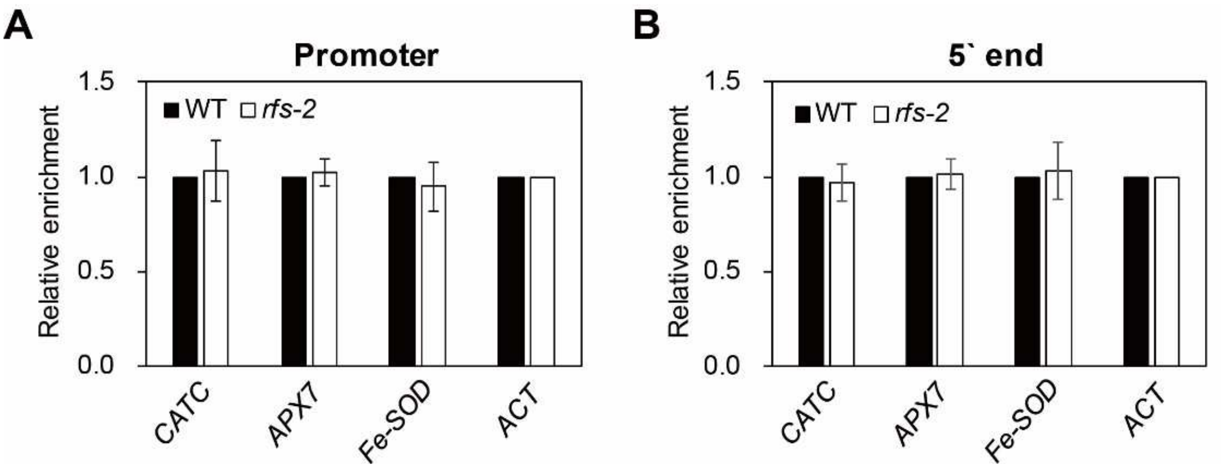

FIGURE S5. Relative enrichment of H3K4me3 on *CATC*, *APX7* and *Fe-SOD* genes in the *rfs-2* mutants.

ChIP analysis of H3K4me3 on three ROS-scavenging genes in the leaves of two-month-old WT and *rfs-2* (see Figure S4). Enrichment of H3K4me3 on the promoter **(A)** and the 5′ end region **(B)** of the three ROS-scavenging genes was measured by ChIP followed by qPCR. *OsActin7* (*ACT*) was used for the normalization of the qPCR analysis. Means and standard deviations were obtained from three biological replicates.

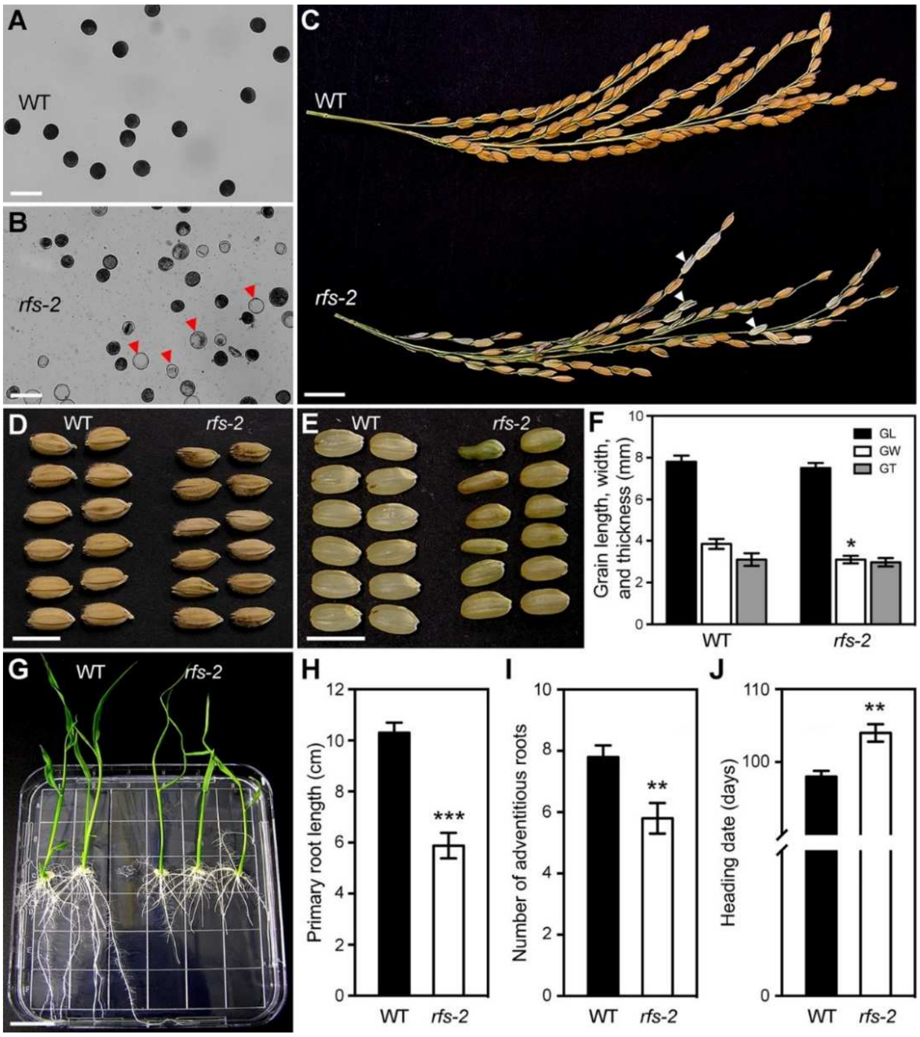

FIGURE S6. The *rfs-2* mutants exhibit multiple development defects.

(A, B) Viability of WT and *rfs-2* pollens. Pollen grains were stained with I2-KI solution. Pollen grains staining black were judged as viable, and those staining grey or light grey (red arrow) were considered sterile. **(C)** Sterilized seed of the *rfs-2* panicles. **(D-F)** Altered seed development and maturation of the *rfs-2*. Grain width was reduced development. GL, grain length; GW, grain width; GT, grain thickness. **(G-I)** Abnormal root development of the *rfs-2* seedlings. Phenotypic characterization and measurement analysis showed that the *rfs-2* had shortened primary roots and reduced numbers of adventitious root. **(J)** Heading date in WT and *rfs-2*. Asterisks indicate statistically significant differences compared with WT as determined by Student's t-test (*, $P < 0.05$; **, $P < 0.01$; ***, $P < 0.005$). Scale bars: 100 μm **(A, B)**; 2 cm **(C)**; 1 cm **(D, E)**; 1.5 cm **(G)**.
